# Laparoscopic Projection Mapping of the Liver Portal Segment, Based on Augmented Reality Combined With Artificial Intelligence, for Laparoscopic Anatomical Liver Resection

**DOI:** 10.7759/cureus.48450

**Published:** 2023-11-07

**Authors:** Meidai Kasai, Hideaki Uchiyama, Tsukasa Aihara, Shinichi Ikuta, Naoki Yamanaka

**Affiliations:** 1 Department of Surgery, Meiwa Hospital, Nishinomiya, JPN; 2 Graduate School of Science and Technology, Nara Institute of Science and Technology, Ikoma, JPN

**Keywords:** surgery, future technologies in minimally invasive surgery and artificial intelligence, artificial intelligence in surgery, augmented reality surgical navigation, virtual augmented reality, augmented reality, 3d surgical navigation, anatomical liver resection, laparoscopic liver surgery, ai & robotics in healthcare

## Abstract

Hepatocellular carcinoma causes intrahepatic metastasis via the trans-portal vein. Thus, appropriate mapping of portal segments is necessary for laparoscopic anatomical liver resection. However, because of the difficulty in identifying tactile sensations and the limited surgical view of laparoscopy, augmented reality (AR) has recently been utilized in laparoscopic liver surgery to identify the tumor, vessels, and portal segments. Moreover, artificial intelligence (AI) has been employed to identify landmarks in two-dimensional (2D) images because of concerns regarding the accuracy of superimposing a three-dimensional (3D) model onto a 2D laparoscopic image. In this study, we report an AR-based projection mapping method of portal segments superimposing preoperative 3D models assisted by AI in laparoscopic surgery. The liver silhouette in laparoscopic images should be detected to superimpose 3D models. Labeled liver silhouettes were obtained from 380 images in surgical videos as learning images to implement AI-based silhouette detection. To implement this technique, we used Detectron2, a PyTorch-based object detection library by Facebook AI Research (Now, Meta AI, Menlo Park, California, United States). In the videos, the liver edges were displayed as green outlines according to AI. Additionally, 3D liver models with segmental mapping were generated using the open-source software 3D Slicer from computed tomography images. For AR display, we utilized the model target function of Vuforia SDK (PTC, Inc., Boston, Massachusetts, United States), an industrial AR library with silhouette-based AR display. Lastly, we merged the AI output video with a 3D model in Unity (Unity Software Inc., San Francisco, California, United States) to establish the projection mapping of the portal segment on 2D surgical images. The accuracy was assessed by measuring the maximum error between the liver edges of laparoscopic images and 3D liver silhouettes in five surgical videos. The maximum error between liver edges and 3D model silhouettes ranged from 4 mm to 22 mm in the AI-based approach and 12 mm to 55 mm in the non-AI-based approach. Meanwhile, the mean error was 14.5 and 31.2 mm in the AI-based and non-AI-based approaches, respectively. Despite camera movement, 3D AR displays were maintained. Thus, our AI-assisted projection mapping of the portal segment could offer a new approach for laparoscopic anatomical liver resection.

## Introduction

Hepatocellular carcinoma causes intrahepatic metastasis via the trans-portal vein; thus, appropriate liver mapping of portal segments is crucial in laparoscopic anatomical liver resection (LALR) in order to systematically excise the tumor-bearing portal territory [1]. Recently, methodologies utilizing indocyanine green (ICG) technology are increasingly being adopted to facilitate the implementation of positive and negative staining methods, thereby significantly enhancing surgical outcomes [2]. The positive staining method visualizes the tumor-bearing portal territory after injecting ICG into the target portal vein [1-3]. By contrast, the negative staining method involves a systemic ICG injection following the clamping of the Glissonean pedicle that supplies the tumor-bearing segment, leading to the formation of ischemic, non-fluorescent lesions [2]. In 2020, Xu et al. reported success rates of 53% and 51% for the positive and negative staining methods, respectively [4]. Given these relatively low success rates, there is a clear demand for incorporating intraoperative navigation using three-dimensional (3D) technology to complement existing approaches.

Augmented reality (AR) is a digital technology that superimposes 3D models from preoperative images onto surgical fields, thereby enhancing the recognition of vascular structures and liver portal segments by projecting transparent models. Deng et al. reported a case of laparoscopic right anterior sectionectomy utilizing a 3D-model-based AR navigation system integrated with ICG technology [5]. Recently, the use of AR in laparoscopic anatomical resections has been reported to be associated with improved surgical outcomes, including reduced operative blood loss and shorter hospital stays [6].

With the ongoing advancements in AR technology, artificial intelligence (AI) has been increasingly incorporated into the medical field to aid in the identification of anatomical landmarks during surgical procedures. In recent years, a growing number of institutions have synergistically integrated AR navigation and AI, facilitating more precise visualization of anatomical structures [7,8].

In the present research, we introduced an AR-driven methodology for delineating portal segments because of the critical role of segmental mapping of the liver’s portal territory in LALR. This method involves overlaying preoperative 3D models and was further enhanced through AI assistance within the context of laparoscopic surgery.

Parts of this work were presented at the Frontiers of Computer-Aided Surgery Symposium in Tokyo, Japan on November 2, 2022. Further aspects of this research were discussed at the 123rd annual congress of the Japan Surgical Society in Tokyo, Japan on April 28, 2023, and some findings were shared at the 35th meeting of the Japanese Society of Hepato-Biliary-Pancreatic Surgery on July 1, 2023. More of this work was presented at the 78th General Meeting of the Japanese Society of Gastroenterological Surgery on July 13, 2023. This technical report provides a comprehensive compilation of our research and findings that have not been published in their entirety before.

## Technical report

**Figure 1:**
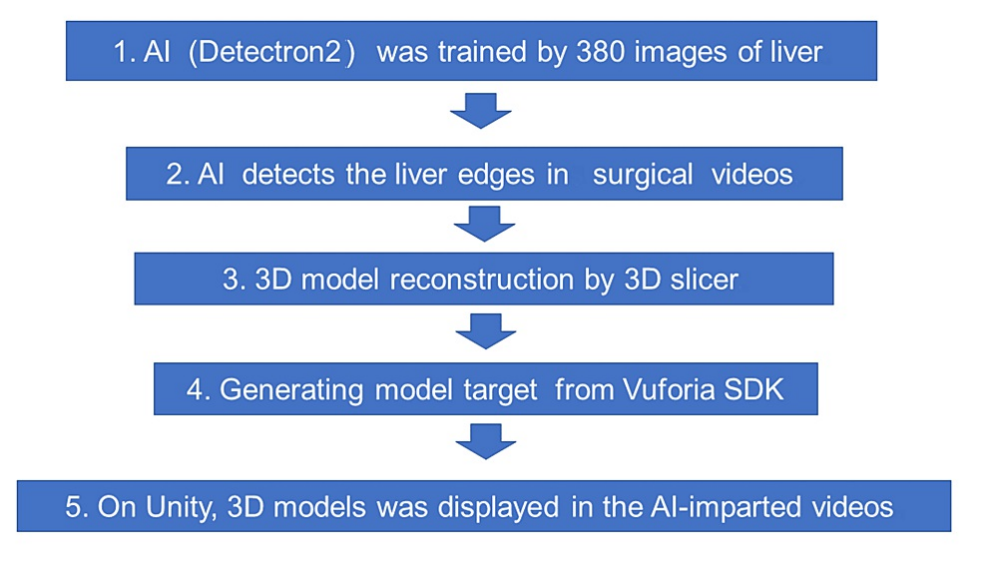
Flowchart of our AI-assisted AR navigation system. The figure illustrates the progression from AI training with liver images to the display of 3D models in AR AR, augmented reality; AI, artificial intelligence Developer/Manufacturer Details: Vuforia SDK (PTC, Inc., Boston, Massachusetts, United States); Unity (Unity Software Inc., San Francisco, California, United States); Detectron2 (Meta AI, Menlo Park, California, United States)

This study was approved by the Medical Ethics Committee of Meiwa Hospital (approval number: 2022-27). All patients provided written informed consent. Figure 1 shows a flow chart of the entire process of our AR-driven methodology.

AI implementation

**Figure 2:**
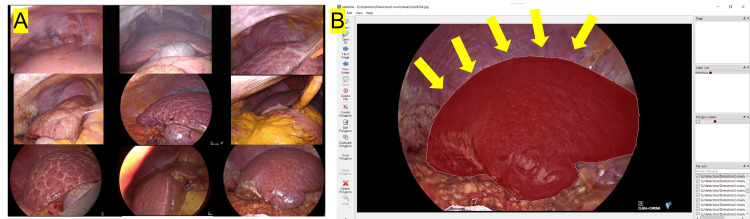
Training datasets with annotations of liver silhouettes. (A) A total of 380 images were extracted from videos of laparoscopic liver surgeries. (B) The liver silhouettes in all images were annotated by experienced surgeons using the LabelMe tool. The yellow arrow indicates the resection border in the images.

Initially, AI (specifically employing the Detectron2 library; Meta AI, Menlo Park, California, United States) was utilized for identifying liver silhouettes in images captured during laparoscopic liver resection. These silhouettes were annotated by experienced hepatobiliary surgeons using LabelMe (GitHub, Inc., San Francisco, California, United States), an open-source graphical image annotation tool (Figure 2) [9].

Detectron2 is a comprehensive open-source object detection library developed by Facebook AI Research (Now Meta AI) [10]. Utilizing models such as panoptic segmentation, mask region-based convolutional neural network (R-CNN), and rotated bounding boxes, it provides robust tools designed for detection and various segmentation tasks. In our work, we leveraged a pretrained mask R-CNN from the Common Objects in Context (COCO) dataset, which was implemented using Python (version 3.8) on a local computer outfitted with an Intel Core i7 (Intel Corporation, Santa Clara, California, United States) and NVIDIA® T1200 Laptop GPU (Nvidia Corporation, Santa Clara, California, United States). Annotated liver images from 380 frames, extracted from laparoscopic surgery videos, were utilized and designated as either training or validation datasets to train the deep CNN model.

3D model reconstruction and model target generation

3D Slicer, an open-source software, was employed to generate a 3D liver model with portal segmental mapping, derived from computed tomography images with a slice thickness of 2 mm [11]. We integrated Unity 2020.3.30 (Unity Software Inc., San Francisco, California, United States) and Vuforia SDK (PTC, Inc., Boston, Massachusetts, United States) for the AR display. While Unity primarily functions as a platform to generate 3D content (e.g., video games or animations), Vuforia SDK offers industrial-standard AR functionalities, enabling the display of 3D models.

**Figure 3:**
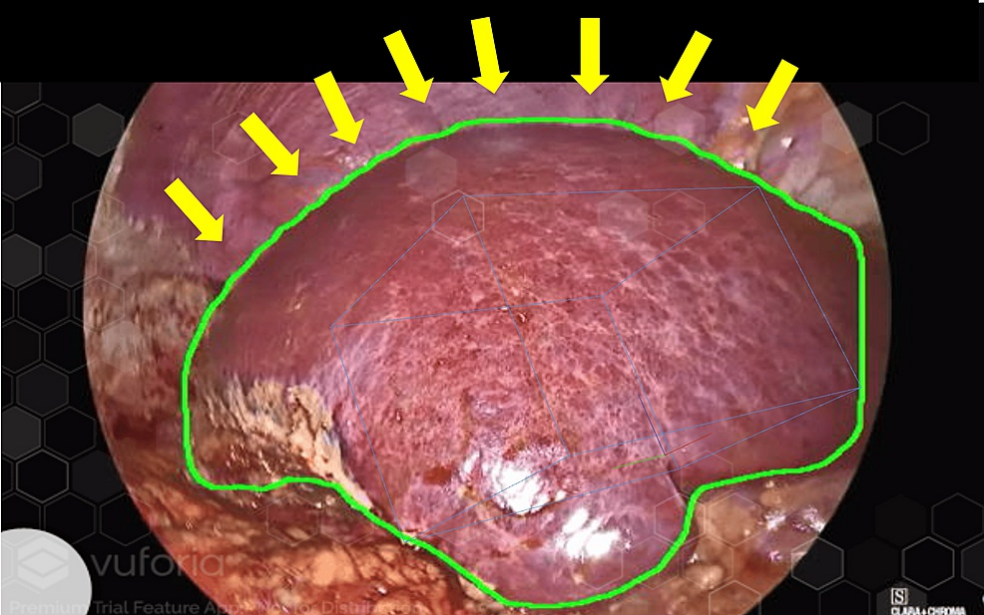
AI-delineated liver edges. Detectron2 (Meta AI, New York, United States), which was trained on liver images, showcases the liver silhouettes outlined in green (indicated by the yellow arrow). AI, artificial intelligence

We utilized a 3D liver model and generated a model target using Vuforia’s Model Target Generator. This feature within the platform supports the rigid transformation of a 3D model with a single camera based on pre-registered models. The engine can identify and track natural silhouettes within an image, provided they remain at least partially visible to the camera. Considering potential obstructions from organs including the kidney, gallbladder, and colon, our registered 3D models comprised only the frontal surface. Models of the right and left lateral liver lobes were used distinctly for individual segmentation. The primary function of the AI system was to delineate liver boundaries, as illustrated by the green lines in Figure 3.

We presented 3D liver models with portal segmental mapping utilizing AI-enhanced video and compared their accuracy to that of the original video. We identified the maximum error in the starting position, measured in millimeters, to assess the system’s precision. We converted pixel values into millimeters by referencing the dimensions of the 3D liver model. This conversion allowed us to evaluate the error in tangible units of measurement, providing a more insightful understanding of the system’s level of accuracy.

Results

Our AR system was tested using a dataset comprising five surgical scenes, each lasting 20 seconds. In the first case, the original surgical video is displayed in the upper-left quadrant, and the AI-processed video is situated in the lower-left quadrant, showcasing the AI’s detection of liver edges in Video 1. This comparison highlights the robustness of the AI-based method, whereas the non-AI-based method occasionally loses tracking.

**Video 1:** Laparoscopic segmental mappping of rigt liver lobe. AR, Augmented Reality; AI, Artificial Intelligence

Similar to the first case, the AI-based method proficiently identifies the liver silhouette, whereas the non-AI-based method encounters difficulties in Video 2.

**Video 2:** Laparoscopic segmental mapping of segment 8. AR, Augmented Reality; AI, Artificial Intelligence

In the third case, the non-AI-based method inaccurately detects the liver silhouette; however, despite some registration errors, the AI-based method still outperforms the non-AI-based method in Video 3.

**Video 3:** Laparoscopic segmental mapping of segments 4, 5, 8. AR, Augmented Reality; AI, Artificial Intelligence

In the fourth case, although the non-AI-based approach seems to register correctly, the AI-based method exhibits superior accuracy, particularly when the liver silhouette undergoes changes in Video 4.

**Video 4:** Laparoscopic segmental mapping of segment 3. AR, Augmented Reality; AI, Artificial Intelligence

Finally, in the fifth case, the non-AI-based method exhibits inconsistencies in performance, whereas the AI-based method maintains consistent and accurate liver registration in Video 5.

**Video 5:** Laparoscopic segmental mapping of left lateral lobe. AR, Augmented Reality; AI, Artificial Intelligence

The registration error results are summarized in Table 1. The table displays the maximal discrepancy between the liver’s edges and the silhouette of the 3D model. Errors range from 4 mm to 22 mm for the AI-based method and 12 mm to 55 mm for the non-AI-based method. The mean error was 14.5 and 31.2 mm for the AI-based and non-AI-based methods, respectively.

**Table 1:** Registration errors in all five cases. This table illustrates the maximum registration errors between the liver’s edges and the 3D model’s silhouette comparing the discrepancies in AI-assisted versus non-AI-assisted AR. AI, artificial intelligence; AR, augmented reality

Case number	Location	Maximum error, mm	
		AI-assisted AR	Non AI-assisted AR
1	Right liver	4	17
2	Right liver	19	48
3	Right liver	14.5	55
4	Left lateral liver	22	12
5	Left lateral liver	13	24
Mean		14.5	31.2

## Discussion

The precision of image-to-patient registration is vital for the efficacy of AR navigation systems. Many studies have reported enhanced results with AR systems that incorporate additional equipment, such as stereo cameras or specialized positional tracers [12-15]. However, a monocular camera is typically the standard equipment in contemporary operating rooms. Therefore, an AR navigation system that operates optimally within a monocular setting needs to be developed [16].

In the context of a monocular camera environment, silhouettes or anatomical landmarks of the liver, including ligaments, are frequently utilized for registration purposes. In 2017, Koo et al. reported a system that employs 3D to two-dimensional (2D) silhouette-based registration for laparoscopic liver surgery [16]. This system is semi-automated, necessitating surgeons to manually mark curvilinear anatomical landmarks. In 2022, Koo et al. further refined their approach by introducing a 3D-to-2D AI-assisted contour-based registration system capable of automatically detecting anatomical landmarks. However, they observed significant variability in the accuracy of AR displays generated from a monocular camera using AI, with errors ranging from 0 mm to 250 mm [8].

Similarly, Labrunie et al. reported a U-net-based deep-learning registration method that initiated registration with tumor target registration errors ranging from 7.2 mm to 22.4 mm [7]. By contrast, a tracer-based registration method developed by Pelanis et al. yielded registration errors between 3.78 mm and 9.75 mm, and a stereo-camera-based iterative closest point (ICP) registration method developed by Luo et al. obtained registration errors ranging from 6.04 mm to 8.73mm [15,17]. The latter two approaches demonstrated smaller registration errors compared with the AI-based approach.

While AI technology eliminates the need for specialized sensors and tracers, its accuracy needs to be further improved [8]. Nonetheless, the automated detection of liver landmarks is invaluable as it minimizes additional tasks required by surgeons, thereby streamlining the registration process.

In laparoscopic liver surgery, the impact of pneumoperitoneum on the liver silhouette is notably significant. Various strategies have been advanced to counter liver deformation in order to mitigate this effect, including locally rigid registration [18], biomechanical simulations for 3D-to-2D rigid registration utilizing the finite element method [19], ICP deformable registration with a stereo camera [15], and contour-oriented deformable registration [8]. The scholarly articles based on these methodologies necessitate a profound understanding of computational graphics and complex C++ coding.

Our proposed method, which was executed within the Unity and Vuforia SDK environments, is relatively straightforward to establish, owing to its low-code structure that eliminates the need for extensive programming. This simplicity is advantageous, facilitating easy deployment in standard operating rooms. Although Vuforia SDK is user-friendly and easy to implement, it provides only rigid registration. This limitation may be significant, especially considering the dynamic deformation of the liver, which can lead to registration errors. Our approach takes cues from the locally rigid registration paradigm proposed by Song et al., which posits that local rigid registration within a small region of interest is sufficient for image guidance without deformable modeling [18]. For instance, models of the right and left liver lobes are utilized exclusively for their respective segmentation. Nonetheless, this method still needs to be improved to better account for organ deformation.

Finally, aside from the limitations that were mentioned previously, our method possesses additional constraints. It is applicable solely prior to the dissection phase as significant alterations to the liver’s shape during dissection render object tracking ineffective. Moreover, interference may arise because of the use of surgical instruments (e.g., forceps) and echo sonography. Consequently, future iterations of AI and AR systems must be sufficiently robust to navigate and mitigate such challenges effectively.

## Conclusions

The application of AI has proved beneficial for the projection mapping of liver portal segments in laparoscopic liver surgery, which is achieved via AR superimposition. This approach can serve as a valuable supplementary technique, particularly for patients wherein ICG segmental mapping is challenging.

For the successful integration of our proposed method into clinical practice, physiological organ deformation should be considered, and potential obstacles (e.g., interference from surgical instruments and liver shape changes induced by hepatic dissection) should be accounted for. These considerations are crucial to improve the technique and ensure its robustness in the dynamic surgical environment.
